# Mechanism of action of 5-arninosalicylic acid

**DOI:** 10.1155/S0962935192000243

**Published:** 1992

**Authors:** N. A. Punchard, S. M. Greenfield, R. P. H. Thompson

**Affiliations:** Gastrointestinal Laboratory The Rayne Institute St Thomas' Hospital London SE1 7EH UK

## Abstract

5-Aminosalicylic Acid (5-ASA) has been used for over 50 years in the treatment of inflammatory bowel disease in the pro-drug form sulphasalazine (SASP). SASP is also used to treat rheumatoid arthritis. However whether the therapeutic properties of SASP are due to the intact molecule, the 5-ASA or sulphapyridine components is unknown. Several mechanisms of action have been proposed for 5-ASA and SASP including interference in the metabolism of arachidonic acid to prostaglandins and leukotrienes, scavenging,of reactive oxygen species, effects on leucocyte function and production of cytokines. However, it is unlikely that the anti-inflammatory properties of SASP and 5-ASA are due to several different properties but more likely that a single property of 5-ASA explains the theraapeutic effects of 5-ASA and SASP. Reactive oxygen species (ROS) are involved in the metabolism of prostaglandins and leukotrienes and can act as second messengers, and so the scavenging of ROS may be the single mechanism of action of 5-ASA that gives rise to its antiinflammatory effects in both inflammatory bowel disease and rheumatoid arthritis.


Mediators of Inflammation, 1, 151–165 (1992)

### Mechanism of action of 5-aminosalicylic acid

N. A. Punchard,[CA] S. M. Greenfield and R. P. H. Thompson

Gastrointestinal Laboratory, The Rayne Institute, St Thomas' Hospital, London SE1 7EH, UK

[CA] Corresponding Author

**5-AMINOSALICYLIC ACID (5-ASA) has been used for over 50 years in the treatment of inflammatory bowel disease in the pro-drug form sulphasalazine (SASP). SASP is also used to treat rheumatoid arthritis. However whether the therapeutic properties of SASP are due to the intact molecule, the 5-ASA or sulphapyridine components is unknown. Several mechanisms of action have been proposed for 5-ASA and SASP including interference in the metabolism of arachidonic acid to prostaglandins and leukotrienes, scavenging of reactive oxygen species, effects on leucocyte function and production of cytokines. However, it is unlikely that the anti-inflammatory properties of SASP and 5-ASA are due to several different properties but more likely that a single property of 5-ASA explains the therapeutic effects of 5-ASA and SASP. Reactive oxygen species (ROS) are involved in the metabolism of prostaglandins and leukotrienes and can act as second messengers, and so the scavenging of ROS may be the single mechanism of action of 5-ASA that gives rise to its anti-inflammatory effects in both inflammatory bowel disease and rheumatoid arthritis.**

**Key words:** 5-Aminosalicylic acid, Inflammatory bowel disease, Reactive oxygen species, Rheumatoid arthritis, Sulphasalazine

### Introduction

Although corticosteroids are the major anti-inflammatory agents used to treat the chronic inflammatory conditions rheumatoid arthritis (RA) and inflammatory bowel disease (IBD), another useful agent is the salicylate derivative 5-aminosalicylic acid (5-ASA). 5-ASA has been used for over half a century in the pro-drug form sulphasalazine (SASP) to treat ulcerative colitis (UC), but was originally designed for use in RA, in which, until relatively recently, it has not been widely used. More recently, SASP has also been used in the treatment of mild psoriasis.[1]

In the late 1930s Dr Nanna Svartz of the Karolinska Institute, Stockholm, became interested in treating RA, believed then to be due to an infectious agent, with the recently developed anti-bacterial agents, the sulphonamides, which were beneficial in the treatment of septic arthritis.[2] However, treatment with these agents either alone or in conjunction with salicylates, which were already used to treat RA, proved ineffective. As salicylate drugs reduced the swelling in inflamed joints but had a lesser effect in reducing inflammation elsewhere in the body it was possible that they became concentrated in the joints, and thus Dr Svartz had the concept that if the salicylate was bound to the sulphonamide then the salicylate might carry it into the joint, where it would be antibacterial. Dr Svartz therefore tried to bond salicylic acid chemically to sulphapyridine, but without success.

However, as a result of a chance meeting she convinced Pharmacia in Stockholm to help[2] and in collaboration with their chemists, E. Askelof and Dr P. H. Willstaedt, a variety of different combinations of salicylate and sulphonamide were produced. One of these, salicylazosulphapyridine, more commonly referred to as sulphasalazine (SASP) and consisting of 5-aminosalicylic acid (5-ASA) and sulphapyridine (SP) joined together by a diazo bond, proved to be effective in treating RA.

In collaboration with Sture Helander, SASP was shown to have an affinity for connective and elastic tissue, such as in the joint capsules and in the bowel, to the extent of forming deposits that gradually broke down to produce 5-ASA and SP.[3–5] As in UC the inflammatory changes occurred in the sub-epithelial connective tissue and as SP had been used with moderate success by Dr Svartz in the treatment of UC, which was then also believed to be due to an infectious agent,[6,7] she treated UC patients with SASP and found that "some cases which did not become free of symptoms with sulphapyridine rapidly improved with salazopyrine".[6]

However, the Second World War and the development of more effective gold based drugs in

© 1992 Rapid Communications of Oxford Ltd the treatment of RA[8] retarded the use of SASP in UC and RA. It was therefore not until the early 1950s that SASP was used in the USA[9] and the UK, and in 1963 the first controlled, double-blind trials showed SASP to be effective in the treatment of mild UC,[10] and later in maintaining remission of UC.[11] In the 1970s its use for RA was rediscovered[12] and confirmed in a series of studies.[13]

In 1977 direct application of the components of SASP to the colonic mucosa in patients with acute UC demonstrated that 5-ASA, but not SP, produced remission in UC similar to that achieved with SASP,[14] as confirmed in other studies,[15,16] and that SP was relatively inactive.[17] Thus it is now considered that in the treatment of UC the SASP acts as a pro-drug, with SP acting as a carrier, delivering the active moiety, 5-ASA, to the inflamed colon. The realization that 5-ASA is the active moiety and that the SP component is responsible for the side effects of SASP, such as infertility, haemolytic anaemia, photosensitization and agranulocytosis, has led to the development of new preparations of 5-ASA. These consist of 5-ASA alone, either covered by a pH sensitive coating or as slow release microgranules that break down in the colon, or of 5-ASA bound to an inactive carrier molecule or to another molecule of 5-ASA by an azo bond (azodisalicylic acid) that, as for SASP, is broken down by bacteria releasing the 5-ASA in the bowel. The main use of 5-ASA in IBD is in UC, although it has some benefit in the treatment of patients with the IBD Crohn's disease (CD).

In RA, although some studies have indicated that SP may be the active moiety[18–21] due to a bacteriostatic effect, others show that SP on its own is ineffective[22] and it has been suggested that the intact SASP molecule may possess anti-inflammatory properties.[23–25] The ability of SASP to accumulate in connective tissues of the joints where it is broken down to release 5-ASA, raises the possibility that SASP may act also as a pro-drug in RA, but delivering 5-ASA, the active component, to the inflamed joint where it is slowly released. The lack of effect of oral 5-ASA in RA can be explained on the basis of its rapid metabolism and excretion once absorbed from the gastrointestinal tract.

Despite its use for over 50 years, the mechanism of action of SASP and 5-ASA remains unclear. A large number of studies have lead to a wide range of hypotheses. This review will attempt to relate the effects of SASP and 5-ASA on production of biological mediators to their effects on cells and tissues, and to those of nonsteroidal anti-inflammatory agents, but is mainly concerned with the proposed mechanisms of action in IBD, from which comes most of the information available.

### Arachidonic Acid Metabolism

Enzymatic oxidation of the essential fatty acid arachidonic acid gives rise to three main groups of compounds: the prostaglandins (PGs) and thromboxanes (TXs) produced by the cyclooxygenase pathway; the leukotrienes (LTs) and the intermediate hydroxyeicosatetraenoic acids (HETEs) from the lipoxygenase pathway; and another group, the lipoxins. The lipoxins have not been studied with respect to SASP and 5-ASA. The products of arachidonic acid are rapidly turned over, locally acting mediators that have been implicated in a range of physiological procedures including reproduction, inflammation, immunological responses and cell growth, division, motility and transport processes.

*The cyclooxygenase pathway:* Early studies indicated that some PGs, for example prostaglandin $E_2$ ($PGE_2$) were proinflammatory. Thus the increased levels found at sites of inflammation or in samples derived from inflamed sites, and the increased PG production by leucocytes from patients with chronic inflammatory diseases all supported this. Raised concentrations of prostaglandins in the stools of patients with active UC were first described by Gould[26] and subsequently increased concentrations of $PGE_2$ were found in rectal mucosal biopsy specimens,[27–31] stools[32,33] and rectal dialysates of UC patients[34–37] with active disease, suggesting that the increased PGs were associated with generation of the inflammation in IBD. The non-steroidal anti-inflammatory agents (NSAIDs) are considered to act through inhibition of the cyclooxygenase pathway, and so increased PG production by the inflamed bowel on removal of SASP treatment or decreased production with SASP[27,28,34,36] or 5-ASA[35,37] suggested that the mechanism of action of SASP and 5-ASA was also through inhibition of the cyclooxygenase enzyme.

Earlier *in vitro* studies supported this, but later ones do not (Tables 1 and 2). Although there are exceptions, we[38] and others[39–46] have demonstrated that relatively low concentrations of SASP and/or 5-ASA can enhance PG production with inhibition of PG production mainly only occurring at high concentrations. Even in some studies in which PG production was only inhibited, promotion can be observed either as a negative inhibition[41] or by a dose related, but not significant, enhancement.[40] Although SP enhanced production of one out of four PGs measured in one study,[41] in the others, either in cell free incubations,[33,41,43,47,48] or in incubations of gastrointestinal biopsies[27,30] or mononuclear (MN) cells,[38] SP either had no effect[33,36,40,48] or inhibited[27,30,31,38,39,41,47,49] PG production, dose dependently.[38]

Except in one study where 5-ASA actually

**Table 1.** *In vitro* studies of the effects of sulphasalazine on prostaglandin and thromboxane production

| Study | Tissue studied | Concentration mol $l^{-1}$ | Effects on cyclooxygenase products |
|---|---|---|---|
| Collier *et al.*; 1976[47] | Bull seminal vesical homogenates | $IC_{50} = 4.7 \times 10^{-4}$ | $\downarrow(E_2 + F_{2\alpha})^{\#}$ |
| Smith *et al.*; 1978[27] | Human normal and inflamed mucosal homogenates | $0.2\text{–}50 \times 10^{-4}$ | $\downarrow(I_2 + F_{2\alpha})$ |
| Sharon *et al.*; 1978[31] | Human normal and inflamed rectal biopsies | $2.5 \times 10^{-4}$ | $\downarrow E_2$ |
| Hoult & Moore; 1978[49] | Rat colonic microsomes | $10^{-8}\text{–}10^{-3}$ | $\downarrow E_2$ |
| Hoult & Moore; 1980[48] | Animal microsomal preparation | $0.1$ & $1 \times 10^{-3}$ | $\downarrow(E_2 + F_{2\alpha})$ |
| Ligumsky *et al.*; 1981[30] | Human normal and inflamed rectal biopsies | $2.52 \times 10^{-4}$ | $\downarrow I_2$, $\downarrow E_2$, $\downarrow TXA_2$ |
| Gould *et al.*; 1981[33] | Rat fundus strip (indirect bioassay) | $0.2\text{–}2 \times 10^{-3}$ | $\downarrow$PG |
| Schlenker & Peskar; 1981[52] | Human colon mucosal microsomes | $2.5 \times 10^{-4}$ | $\downarrow\uparrow E_2^{d}$ |
| Rachmilewitz *et al.*; 1982[40] | Unstimulated PBMNC from healthy subjects | $1.26$ & $2.52 \times 10^{-4}$ | NA. |
| | | $5 \times 10^{-4}$ | §($\uparrow^{a}$) $E_2$, $\downarrow TXA_2$ |
| Hawkey & Trulove; 1983[41] | Colitic rectal biopsy homogenates | $10^{-6}$ & $10^{-4}$, | $\uparrow E_2^{c}$ |
| | | $10^{-3}\text{–}10^{-2}$ | $\downarrow E_2$ |
| Stenson & Lobos; 1983[42] | Platelets | $0.1\text{–}4.0 \times 10^{-3}$ | $\uparrow E_2$, $\uparrow F_{2\alpha}$, $\downarrow TXA_2$ |
| Hawkey *et al.*; 1985[43] | Human colonic mucosal homogenates | $5 \times 10^{-5}$ | §$I_2$, §$E_2$, $\uparrow F_{2\alpha}$, $\downarrow TXA_2$ |
| | | $10^{-3}$ | $\downarrow I_2$, §$E_2$, $\uparrow F_{2\alpha}$, $\downarrow TXA_2$ |
| Kolassa *et al.*; 1985[45] | Rabbit colonic microsomes | $10^{-3}$ | NA on $I_2$, $E_2$, $F_{2\alpha}$, $TXA_2$ |
| Hillier *et al.*; 1984[134] | Stimulated PBMNC | $1.26 \times 10^{-4}$ | $\uparrow E_2$ |
| Keating *et al.*; 1988[46] | Stimulated PBMNC from IBD patients | (not quoted) | $\uparrow E_2$ |
| Punchard *et al.*; 1992[38] | PBMNC from healthy subjects; Unstimulated | $10^{-7}\text{–}10^{-5}$ | NA |
| | | $10^{-4}$ | $\uparrow I_2$, $\uparrow E_2$, $\uparrow F_{2\alpha}$ |
| | | $10^{-3}$ | §$I_2$, $\uparrow E_2$, $F_{2\alpha}$ |
| | LPS-stimulated | $10^{-7}$ & $10^{-6}$ | NA |
| | | $10^{-5}$ & $10^{-4}$ | $\uparrow I_2$, $\uparrow E_2$, $\uparrow F_{2\alpha}$ |
| | | $10^{-3}$ | $\downarrow I_2$, §$E_2$, $\downarrow F_{2\alpha}$ |

$E_2$ = Prostaglandin $PGE_2$; $F_{2\alpha} = PGF_{2\alpha}$; $I_2 = PGI_2$; $TXA_2$ = Thromboxane $A_2$; PBMNC = peripheral blood mononuclear cells.

In these studies $PGI_2$ and $TXA_2$ were measured as their respective stable hydrolysis products $6KF_{1\alpha}$ and $TXB_2$.

§ = no effect; $\uparrow$ = enhancement; $\downarrow$ = inhibition. NA = no effect on any PGs measured, as opposed to those PGs measured as stated which were effected.

### In this study the sum of both PGs was reported.

[a] Enhancement in this study was not significant but as it is large and increased with increasing concentration of drug we have included it.

[c] In this study results are presented as % inhibition and this enhancement is apparent as negative (<0%) inhibition which is reduced with increasing concentration to produce inhibition at high concentrations.

[d] In this study whether PG production was enhanced or inhibited depended on the concentration of substrate used.

All concentrations have been converted to mol $l^{-1}$ to allow direct comparison.

enhanced production, both SASP and 5-ASA inhibit production of thromboxane $A_2$ ($TXA_2$), a potent vasoconstrictor that increases the adhesion to the endothelium and aggregation of platelets and polymorphonuclear cells (PMN). SP either inhibits[30,40,41] or has no effect[42] on $TXA_2$ production. Although inhibition of $TXA_2$ production by SASP is associated with increased PG production,[42,50] inhibition of $TXA_2$ synthesis by 5-ASA[42] and specific $TXA_2$ inhibitors[42,50] is not, suggesting that the promotion of PGs is not just a result of inhibition of $TXA_2$ synthesis increasing the availability of substrate. However, the converse, that increased synthesis of PGs removes substrate for $TXA_2$ synthesis, may be true. In a model of colitis 5-ASA and inhibitors of $TXA_2$ were equally effective anti-inflammatory agents, suggesting that the anti-inflammatory properties might be through inhibition of $TXA_2$ production.[51]

Differences in the incubation conditions used may alter the responses of the tissues to the drugs and this may be why 5-ASA and SASP inhibit PG production in some studies and, at the same concentration, enhance production in others. For example, in one study the concentration of the exogenous substrate (arachidonic acid) determined whether SASP enhanced or inhibited $PGE_2$ production,[52] while in another addition of substrate reversed the inhibition of MN cell PG production by SASP.[53] We have also found that stimulation of MN cells increases their sensitivity to the effects of SASP, 5-ASA and SP, which may result from increased concentrations of free arachidonic acid released by phospholipases activated by stimulation

**Table 2.** *In vitro* studies of the effects of 5-aminosalicylic acid on prostaglandin and thromboxane production

| Study | Tissue studied | Concentration mol $l^{-1}$ | Effects on cyclooxygenase products |
|---|---|---|---|
| Collier *et al.*; 1976[47] | Bull seminal vesical homogenates | $IC_{50} = 7.1 \times 10^{-3}$ | $\downarrow(E_2 + F_{2\alpha})$# |
| Sharon *et al.*; 1978[31] | Human normal and inflamed rectal biopsies | $3.2 \times 10^{-4}$ | $\downarrow E_2$ |
| Hoult & Moore; 1980[48] | Animal microsomal preparation | $0.1$ & $1 \times 10^{-3}$ | $\downarrow(E_2 + F_{2\alpha})$ |
| Hoult & Page; 1981[39] | Human normal colonic fragments | $5$ & $50 \times 10^{-5}$ | $\uparrow I_2$, $\uparrow F_{2\alpha}$ |
| | | $5 \times 10^{-3}$ | NA |
| | Rat caecal fragments | $5 \times 10^{-5}$ | NA |
| | | $5 \times 10^{-4}$ | $\uparrow I_2$, $\uparrow F_{2\alpha}$ |
| | | $5 \times 10^{-3}$ | $\downarrow I_2$, $\downarrow F_{2\alpha}$ |
| Ligumsky *et al.*; 1981[30] | Human normal and inflamed rectal biopsies | $3.2 \times 10^{-3}$ | $\downarrow I_2$, $\downarrow E_2$, $\downarrow TXA_2$ |
| | | $6.5 \times 10^{-4}$ | §$I_2$, $\downarrow E_2$, $\downarrow TXA_2$ |
| | | $1.3 \times 10^{-3}$ | $\downarrow I_2$, $\downarrow E_2$, $\downarrow TXA_2$ |
| Gould *et al.*; 1981[33] | Rat fundus strip (indirect bioassay) | $0.1–10 \times 10^{-3}$ | $\downarrow$PG |
| Rachmilewitz *et al.*; 1982[40] | Unstimulated PBMNC from healthy subjects | $3.3 \times 10^{-5}$ | NA |
| | | $3.3 \times 10^{-4}$ | $\downarrow E_2$, §$TXA_2$ |
| | | $4.9$ & $32.7 \times 10^{-4}$ | $\downarrow E_2$, $\downarrow TXA_2$ |
| Hawkey & Trulove; 1983[41] | Colitic rectal biopsy homogenates | $10^{-5} - 10^{-3}$ | §$E_2$ |
| | | $10^{-3}$–$10^{-1}$ | $\downarrow E_2$ |
| Stenson & Lobos; 1983[42] | Platelets | $0.1–4.0 \times 10^{-3}$ | §$E_2$, §$F_{2\alpha}$, $\downarrow TXA_2$ |
| Hawkey *et al.*; 1985[43] | Human colonic mucosal homogenates | $5 \times 10^{-5}$ | NA |
| | | $1 \times 10^{-3}$ | §$I_2$, $\downarrow E_2$, §$F_{2\alpha}$, §$TXA_2$ |
| Berry & Hoult; 1984[44] | Rat caecal fragments | $5 \times 10^{-4}$ | $\uparrow I_2$, |
| | | $>5 \times 10^{-4}$ | $\downarrow I_2$ |
| Kolassa *et al.*; 1985[45] | Rabbit colonic microsomes | $10^{-5}$ | $\uparrow I_2$, §$E_2$, §$F_{2\alpha}$, $\uparrow TXA_2$ |
| | | $10^{-4}$ | $\uparrow I_2$, $\uparrow E_2$, §$F_{2\alpha}$, $\uparrow TXA_2$ |
| | | $10^{-3}$ | $\uparrow I_2$, $\uparrow E_2$, $\uparrow F_{2\alpha}$, $\uparrow TXA_2$ |
| Keating *et al.*; 1988[49] | Stimulated PBMNC from IBD patients | (not quoted) | $\uparrow E_2$ |
| Mahida *et al.*; 1991[138] | Human, inflamed biopsy | $3.3$ & $6.6 \times 10^{-4}$ | §$TXA_2$ |
| Punchard *et al.*; 1992[38] | PBMNC from healthy subjects: | | |
| | Unstimulated | $10^{-7}, 10^{-6}, 10^{-5}, 10^{-4}$ | NA |
| | | $10^{-3}$ | $\downarrow I_2$, $\downarrow E_2$, $\downarrow F_{2\alpha}$ |
| | LPS-stimulated | $10^{-7}$ | NA |
| | | $10^{-6}$ | §$I_2$, $\uparrow E_2$, $\uparrow F_{2\alpha}$ |
| | | $10^{-5}$ | §$I_2$, §$E_2$, $\uparrow F_{2\alpha}$ |
| | | $10^{-4}$ | NA |
| | | $10^{-3}$ | $\downarrow I_2$, $\downarrow E_2$, $\downarrow F_{2\alpha}$ |

$E_2$ = Prostaglandin $PGE_2$; $F_{2\alpha} = PGF_{2\alpha}$; $I_2 = PGI_2$; $TXA_2$ = Thromboxane $A_2$; PBMNC = peripheral blood mononuclear cells.
In these studies $PGI_2$ and $TXA_2$ were measured as their respective stable hydrolysis products $6KF_{1\alpha}$ and $TXB_2$.
§ = no effect; $\uparrow$ = enhancement; $\downarrow$ = inhibition. NA = no effect on any PGs measured, as opposed to those PGs measured as stated which were effected.
### In this study the sum of both PGs was reported.
[a] Enhancement in this study was not significant but as it is large and increased with increasing concentration of drug we have included it.
[c] In this study results are presented as % inhibition and this enhancement is apparent as negative (<0%) inhibition which is reduced with increasing concentration to produce inhibition at high concentrations.
[d] In this study whether PG production was enhanced or inhibited depended on the concentration of substrate used.
All concentrations have been converted to mol $l^{-1}$ to allow direct comparison.

with LPS. The increase in production of PG by MN cells from healthy subjects with 5-ASA[38,54] is much less than that found using MN cells from IBD patients.[46] This may indicate that cells from IBD patients are more sensitive to 5-ASA, possibly due to prior activation by the inflammatory process *in vivo*. Some *in vitro* incubations may thus have used relative proportions of SASP, substrate and enzyme that only favoured inhibition. The conversion of added radiolabelled arachidonic acid to PGs, as used in some studies, may not have mimicked the unlabelled endogenous substrate and a low conversion substrate could even result from increased competition and conversion of endogenous substrate.

There is indeed little evidence that SASP and 5-ASA inhibit PG synthesis in patients. In some studies PG production *in vivo* in patients who relapsed during maintenance therapy with SASP increased despite treatment[34] and in patients treated with 5-ASA but who failed to respond to therapy PG levels increased,[37] suggesting that disease activity rather than treatment produces the changes in PG production. Removal of SASP therapy in patients with inactive disease did not affect production.[55] The *in vivo* and *ex vivo* changes observed in patients associated with SASP or 5-ASA therapy may equally have been due to a reduction in the numbers of PG-producing cells in the bowel, specifically the mononuclear cells (MN) since polymorphonuclear cells produce little or no PG.[56] The degree of inflammatory cell infiltrate in animals[55,57] and the increased levels of the substrate arachidonic acid in the mucosa in IBD[58] have been correlated with the cell density of the inflammatory infiltrate[59] and thus changes in PG production may be the secondary consequence of the invasion of the inflamed bowel by MN cells.[28] Changes in permeability with inflammation could also lead to increased leakage of PGs into the bowel. Thus it is likely that the decrease in PG production associated with SASP reflects only a decrease in the number of PG-producing MN cells present in the colonic mucosa during the healing process.

Indomethacin and other (NSAIDs) are far more potent inhibitors of PG production than either SASP or 5-ASA and are not effective in the treatment of, and may even exacerbate, UC.[60–64] Moreover, not only can PGs and their analogues protect the gastric and small intestinal mucosa against injury (cytoprotection) but they are also protective in the colon.[65–69] Production of PGs may therefore be beneficial rather than damaging. A failure to produce protective PGs in response to stimulation in the inflamed bowel may exacerbate the disease and be important in maintaining the inflammatory process. However, in RA NSAIDs are beneficial. This would suggest that either there are different mechanisms involved in the inflammation in RA, that the site of the inflammation modifies the action of the drugs, or that the effects of NSAIDs are not directly related to their actions on the cyclooxygenase enzyme.

The promotion of PG production with low concentrations of 5-ASA may be because 5-ASA, acting as an antioxidant and reducing co-factor, prevents free radical mediated inactivation of cyclooxygenase.[39,44] Similarly, SASP (see below) can also act as an antioxidant and could thus also act as a reducing co-factor in PG synthesis. Conversely, the inhibition of PG production at high concentrations of SASP and 5-ASA may be due to inactivation of cyclooxygenase by free radical intermediates of the drug.[70] Other antioxidants can also affect PG synthesis,[71–76] and thus some of the inconsistencies between previous *in vitro* studies may be due either to the addition to the incubation media of the antioxidants adrenaline and reduced glutathione,[27,41,47] both of which affect PG production,[41,42,49,72] or to losses of endogenous antioxidants during preparation of the homogenates.[77]

SASP inhibits the NAD-dependent 15-hydroxy PG dehydrogenases (NAD-PGDH), which are enzymes that catabolize PGs to their 15-keto-13,14 dihydro derivatives, in cell free systems,[48,49,78–80] the isolated perfused rat lung[48] and when perfused into rats.[48] NSAIDs such as indomethacin, which are ineffective or deleterious in IBD, also inhibit NAD-PGDH non-competitively,[48,78,80–82] although unlike SASP, they are more active as inhibitors of PG synthesis than breakdown.[49,80] However, NAD-PGDH is unstable under conditions similar to those used in most of the above *in vitro* studies[81] and the inhibition of PGDH by indomethacin is through formation of an unreactive complex and denaturation of the NADH enzyme *in vitro* rather than a phenomenon of physiological importance.[81,82] It is recommended that inhibition of PGDH observed in aqueous solutions should be confirmed under conditions such that denaturation or inactivation would not occur.[81,82] Such studies have not been performed with SASP and thus it is unclear whether SASP inhibits or simply inactivates NAD-PGDH. Other types of PGDH with different specificities for the different PGs, higher reactivity with cofactors other than NAD,[83] and broad substrate specificities[84] have been described and it is unclear whether SASP has the same effects on all of these.

The human lung is less sensitive to SASP than the rat[85] and the high concentrations of SASP used and removal of the effects of SASP upon the addition of albumin to the perfusate suggests that SASP in the circulation will have little effect upon PG degradation *in vivo*. It is possible that SASP, rather than acting inside tissues, blocks PG uptake.[85,86] The unphysiologically high concentrations of PGs and SASP used, and lack of information on the changes in endogenous, unlabelled pools in some of these studies also raises doubts about the interpretation of the results. When PG synthesis and catabolism have been measured together, inhibition of PG catabolism by SASP is not associated with increased production[45,49] or production of both the PG and its metabolite are enhanced simultaneously.[52] Furthermore 5-ASA, and also SP, were not active as inhibitors of the NAD-PGDH.[48,78,79]

Whether SASP and 5-ASA inhibit or enhance MN cell PG production *in vitro* also depends on the compound, its concentration, which PG is being measured, and whether PG production is stimulated or not. The species of animal used also alters the inhibition of PG production by SASP and 5-ASA.[48] The incubation conditions affect the results seen, and so whether the conditions in the mucosa are accurately reproduced in cell and tissue incubations is unknown. The results from such *in vitro* studies should therefore be extrapolated to the therapeutic mechanisms *in vivo* with caution.

Whereas the concentrations of these drugs in the bowel lumen could favour either enhancement or inhibition of PG production, they are probably much lower in the mucosa where they are likely to enhance PG production. The enhancement of PG production by SASP and 5-ASA, with mainly inhibition by the clinically ineffective SP, and the cytoprotective effects of PGs all suggest that enhancement is a more likely mechanism of action of SASP and 5-ASA than inhibition. Thus SASP and 5-ASA could act therapeutically in IBD by enhancing protective PG production either alone, or together with inhibition of pro-inflammatory products of arachidonic acid.

SASP is poorly absorbed in the colon, and is unlikely to have systemic effects on PG degradation. 5-ASA has little, if any, effect on PGDH[48,78,79] and thus inhibition of PG degradation is unlikely to be the mechanism of action of 5-ASA in IBD. SP also inhibits PG production and the PG-inhibiting NSAIDs are ineffective in IBD, thus inhibition of PG production also seems unlikely as a mechanism of action in IBD. That SP also inhibits $TXA_2$ production and yet is ineffective in IBD may suggest that this is also an unlikely mechanism. Whether this is also true in RA depends upon which component of SASP is the active ingredient and whether NSAIDs possess other anti-inflammatory properties besides inhibition of PG production. Whether the mechanism by which 5-ASA and SASP promote PG production *in vitro* is the same or different is not certain, but given that PGs are protective to the colon, promotion of PG production by 5-ASA could explain the therapeutic properties of 5-ASA and the lack of effectiveness of SP in IBD. Whether promotion of PGs could also be protective in RA is unknown and it is possible that SASP and 5-ASA possess other properties that explain their anti-inflammatory effects in RA.

*Lipoxygenase products:* Much of the cellular damage at sites of inflammation is due to the local actions of activated leucocytes at the inflamed site. The increased recruitment of leucocytes from the peripheral blood into the inflamed site is in part a result of increased production of potent chemotactic lipoxygenase products, for example 5-HETE and $LTB_4$. The anti-inflammatory effects of SASP and 5-ASA could thus be through reducing leucocyte recruitment into the inflamed bowel by inhibiting production of these chemotactic agents.

In cell free systems 5-ASA,[87,88] but not SP,[86] inhibits soyabean lipoxygenase activity, but there is disagreement as to whether SASP does[88] or does not,[89] probably a result of different analytical methods being used in the two studies. In cells both SASP and 5-ASA inhibit the production of HETEs by PMN[90–93] and also sulphidopeptide leukotrienes by rat peritoneal cells,[94] but in platelets both these drugs enhance 12-HETE production.[42] SP has either a weak[93] or no effect on the lipoxygenase pathway.[42,87,94] SASP inhibits production of HETEs by inflamed mucosa *in vitro*,[43,94] and both SASP and 5-ASA inhibit synthesis of $LTB_4$ and sulphidopeptide leukotrienes by both normal and inflamed mucosa[96–98] and in rat basophil leukaemia cells,[99] probably through inhibition of glutathione transferase.[99] Although 5-ASA inhibited production of HETEs by inflamed mucosa *in vitro* in one study,[33] it did not in another.[43] Similarly, although acetyl 5-ASA was as good an inhibitor of soyabean lipoxygenase as 5-ASA,[87] other studies show acetyl 5-ASA to have no effect on PMN lipoxygenase activity.[91,92] It is possible that the methods used for determining the activity of the lipoxygenase pathway or, as for PG production, differences in the incubation conditions may alter the result obtained, suggesting that these *in vitro* results should be correlated to the *in vivo* situation with caution.

The promotion of PG production, for example as observed in our MN cell incubations,[38] could have been due to inhibition of the lipoxygenase pathway and redirecting of substrate into prostaglandin synthesis. However, 5-ASA or SASP did not affect MN cell $LTB_4$ production when PG synthesis was enhanced[46] and inhibition of the lipoxygenase pathway was not associated with increased cyclooxygenase activity.[95,97]

How closely these *in vitro* studies are related to the clinical mechanism of 5-ASA and SASP is unclear. Acetyl 5-ASA does not affect IBD, and yet in one study was as good an inhibitor of soyabean lipoxygenase as 5-ASA.[87] Conversely, 4-ASA, as effective as 5-ASA in the treatment of UC, does not inhibit PMN lipoxygenase activity *in vitro*.[90] A 5-lipoxygenase inhibitor was as effective as 5-ASA in a rat model of colitis[100] and yet benoxaprofen, a putative lipoxygenase inhibitor, was ineffective in UC.[101] Although increased levels of $LTB_4$ occur in the inflamed bowel,[95] the concentrations of $LTB_4$ in the colonic lumen were unchanged during successful 5-ASA treatment of patients with IBD.[37] As for PG production, the changes that occur in $LTB_4$ production with remission induced either by a lipoxygenase inhibitor or 5-ASA in an animal model of colitis were concluded to be due more to a reduction in the numbers of PMN, the major source of $LTB_4$, infiltrating the bowel than due to a direct effect on the lipoxygenase pathway.[100]

An action of 5-ASA and SASP as lipoxygenase inhibitors would explain the clinical effects of these drugs in IBD and RA and the ineffectiveness of SP. However, although lipoxygenase inhibitors reduce $LTB_4$ production in UC[102] they still have to be shown to benefit IBD and RA. Many of the products of the lipoxygenase pathway resemble those generated through non-enzymic attack of arachidonic acid by reactive oxygen species (ROS), while ROS are generated in the lipoxygenase pathway. Thus it may be difficult to differentiate between an effect of SASP and 5-ASA as ROS scavengers and an effect as inhibitors of the lipoxygenase pathway.

### Reactive Oxygen Species

Reactive oxygen species (ROS) are forms of oxygen that are more strongly oxidizing than oxygen itself, and include hydrogen peroxide ($H_2O_2$), lipid peroxides, hypochlorous acid (HOCl) and oxygen-free radical species such as the superoxide and hydroxyl free radicals. Their ability to react with cellular constituents, resulting in damage to proteins, nucleic acids and cell membranes, means that ROS are highly toxic to living systems. Their reactive nature also means that ROS react with molecules close to their origin, the distance migrated being directly proportional to their reactivity. The ability of antioxidants to prevent cellular damage at sites of inflammation and ischaemia suggest that the ROS produced in these situations play an important role in generating the tissue injury.

At sites of inflammation the major source of the ROS are activated PMN, although MN cells also produce ROS. Activated PMN produce antimicrobial and cytotoxic ROS, namely the superoxide radical and $H_2O_2$ and, through the action of the enzyme myeloperoxidase (MPO) on $H_2O_2$ and the chloride ion, release HOCl. The ROS released may also react with suitable transition metals to produce the highly reactive hydroxyl radical. In addition, HOCl inactivates alpha-1-protease, which protects tissues against proteolysis at inflamed sites, and activates collagenase released from PMN. Some of the damage in IBD and RA is also suggested to arise from ischaemia. During periods of ischaemia ATP is broken down to hypoxanthine, and the enzyme xanthine dehydrogenase is converted to xanthine oxidase (XOD), which, upon reperfusion of the tissues, utilizes the oxygen to metabolize the hypoxanthine and in so doing generates the superoxide radical. Furthermore, changes in the endothelium promote PMN endothelial interactions, leading to activation of the PMN and a subsequent burst of ROS production from the PMN.

In cell free systems SASP, 5-ASA and SP all scavenge the hydroxyl radical equally, with rate constants close to the diffusion controlled limits.[102] The products of the attack of 5-ASA by ROS from stimulated leucocytes are similar to those produced by the iron-mediated Fenton reaction and are indicative of a reaction between 5-ASA and the hydroxyl radical.[104] However, neither 5-ASA, SASP nor SP affected hydroxyl radical production in a xanthine oxidase cell free system,[105] and only 5-ASA, out of the three, inhibited the effects of hydroxyl radical production by PMN suggesting that under some conditions the effects of SASP and its metabolites may be negligible. Although the ability of 5-ASA to scavenge the hydroxyl radical *in vitro* may not reflect an ability to act as a hydroxyl radical scavenger in IBD,[106] 5-ASA is as effective as the hydroxyl radical scavenger dimethylsulphoxide (DMSO) in preventing experimentally induced ischaemia/reperfusion gastric injury.[107]

The effects of 5-ASA on the hydroxyl radical may be less important in IBD than the effects on leucocyte MPO-derived HOCl.[108] In a cell free system 5-ASA and SP both inhibited MPO-generated HOCl and had a direct inhibitory effect on the MPO enzyme.[109] SASP inhibits the metabolic burst of PMN that fuels the MPO,[110] and SASP and 5-ASA could act as inhibitors of the MPO enzyme rather than as scavengers of the ROS produced by MPO. Certainly the methods used for detecting ROS in cells often do not distinguish between inhibition of ROS production and scavenging effects. However, others have found inhibition of PMN ROS by SASP and 5-ASA not to be associated with decreased oxygen consumption[111,112] and the products of ROS attack of 5-ASA are found in leucocyte incubations,[104] indicating that inhibition of ROS activity by scavenging does occur in some incubations.

When formyl-methionyl-leucyl-phenylalanine (f-mlp) is used to stimulate PMN, 5-ASA[111] and SASP[111,113] and SP[113] all inhibit MPO dependent chemiluminescence. However, when phorbyl myristate acetate (PMA) has been used to stimulate PMN neither 5-ASA[111] SASP[111,113] nor SP[111] affected, chemiluminescence. This lack of effect may either be because the intracellular site of ROS generated by PMA stimulation is inaccessible to the drugs[111] or to a lack of effect of the drugs against ROS produced by mechanisms that are independent of intracellular calcium release, suggesting that the effects of SASP and 5-ASA are calcium dependent.[113] Thus the effects of SASP and 5-ASA on PMN ROS generation depend on the stimulant used.

However, in other studies 5-ASA was an effective inhibitor of MPO activity in PMA-stimulated PMN[112] and in zymosan-stimulated PMN 5-ASA, but not SP, inhibited chemiluminescence.[105] However, SP was a better inhibitor of MPO induced iodination than 5-ASA.[114] SASP was inactive in both studies.[105,114] In cell free systems SP and 5-ASA inhibited MPO by different mechanisms.[114] Although in one study 5-ASA, but not

SASP nor SP, effectively inhibited production of the superoxide radical by PMN,[105] yet conversely, SASP and SP, but not 5-ASA inhibited the activity in another.[114] Differences in incubation conditions and methodology may modify the effect of 5-ASA and SASP on ROS *in vitro* and account for the differences between these studies.

The ability of SASP and diazosalicylic acid to inhibit the activity of the superoxide radical produced by PMN and to inhibit oxygen uptake by the cells, whereas SP was relatively inactive,[110] suggest that this results from direct inhibition of the enzymes.[110] In the same study SASP and diazosalicylic acid inhibited the activity of XOD-generated superoxide and urate production suggesting that again they acted directly on the XOD enzyme and not on the ROS produced.[110] This may be due to the diazo bond, rather than the 5-ASA structure, found in both molecules, since in a cell free system SASP, but not SP nor 5-ASA, inhibited XOD-generated superoxide activity.[105] Conversely, in another cell free study both 5-ASA and SASP reduced the activity of XOD generated superoxide radical and thus might equally be due to the 5-ASA component.[115] However, in this latter study uric acid production was not altered suggesting that the result was due to a scavenging effect rather than inhibition of the XOD enzyme. It is thus unclear whether in addition to scavenging PMN ROS, SASP and 5-ASA may also inhibit the enzymes involved in their production *in vitro*.

5-ASA and SASP also inhibit the action of peroxides. 5-ASA, and to a lesser extent SASP but not SP, reduce the activity of $H_2O_2$ produced by PMN and xanthine oxidase.[105] Furthermore, we have shown that 5-ASA, and to a lesser extent SASP, but SP only marginally, inhibit production of lipid peroxides in ROS stressed erythrocytes,[54,110] and others that 5-ASA is more effective than SASP, diazosalicylic acid or SP in inhibiting haemoglobin catalysed lipid peroxidation.[117] In other studies 5-ASA, but not SASP nor SP, scavenged (reduced) the organic radical 1,1-diphenyl-2-picrylhydrazyl,[92,118] and thus the anti-inflammatory effects of 5-ASA may be directed at other, organic radical species. 5-ASA, but not SASP nor SP, also protects the protein, alpha-1-antiprotease against inactivation by HOCl.[103]

The ability of 5-ASA and SASP to react with ROS with sufficient affinity enables them to protect cellular constituents against ROS-mediated injury as illustrated by the ability of 5-ASA to protect cells in culture against the superoxide radical and $H_2O_2$[104] and also stimulated PMN.[112] Moreover, in a cellular system 5-ASA and SASP, but not SP, also reduced ROS-induced chemiluminescence in colonic mucosal scrapings, and reduced cytochrome C reduction by colonic crypt cells.[115] This suggests that scavenging of ROS may be more important than any effect on the enzymes involved in their generation.

The ROS scavenging ability of 5-ASA and SASP may be the basis of their anti-inflammatory properties. SASP reduced inflammation in acetic acid induced colitis in an animal model as effectively as did superoxide scavengers.[119] The iron chelator desferrioxamine was without effect[119] and thus it is unlikely that the action of SASP and 5-ASA is through binding to ROS generating transition metals.[106] As both XOD inhibitors and DMSO have limited effectiveness in this model, HOCl rather than the hydroxyl or XOD, was the probable target of the scavenging properties of SASP.[119] However, both 5-ASA and DMSO were anti-inflammatory in a model of ischaemia/reperfusion induced gastric injury, suggesting that 5-ASA was an effective hydroxyl radical scavenger.[107] 5-ASA and SASP, but not SP, protected the bowel against both XOD and bile acid induced ROS damage[115] and also in an experimental model of acute ileitis in rats induced by perfusion of f-mlp where PMN-generated ROS give rise to cellular damage.[109] It would thus appear that the ability of SASP and 5-ASA to react with a wide range of ROS allows them to protect against the damage due to the different ROS produced in different circumstances.

SASP, but not 5-ASA, prevent NSAID generated inflammation,[119] which is also reduced or abolished by free radical scavengers in animals. The lack of effect of 5-ASA in this model was suggested to be due to rapid inactivation of 5-ASA when given orally.[120] Moreover, whereas SASP and 5-ASA, but not SP, directly infused into the colon prevents the bile acid induced loss of DNA and increased cellular turnover, the NSAID indomethacin increased it.[115] Although NSAIDs also scavenge ROS,[121,122] which may account for some of their anti-inflammatory properties, the reaction with ROS may also generate toxic free radical derivatives of the NSAIDs,[123] hence the ability of other antioxidants to suppress the toxicity of NSAIDs *in vivo*. However, there is no evidence that the products of the reaction between ROS and 5-ASA and SASP are toxic, and SASP, like other antioxidants, protects against NSAID damage *in vivo*.

The above studies suggest a role for SASP and 5-ASA as scavengers of ROS. Although *in vitro* they may also have a direct effect on the enzymes producing the ROS in the inflamed bowel it is unlikely that, *in vivo*, they will be absorbed in sufficiently high enough concentrations for this effect to exceed their scavenging properties. Thus the antioxidant effects of SASP and 5-ASA are a more likely anti-inflammatory mechanism of action. The lack of effect of SP as a ROS scavenger in most systems is consistent with its lack of therapeutic properties in IBD. Differences observed between some studies may be a result of methodological differences altering the effects of SASP and 5-ASA, for example in one study 5-ASA could not be tested because it directly reacted with the cytochrome C.[110] Although the products of the reaction between 5-ASA and ROS have been found in faeces of patients with IBD, supporting the concept that 5-ASA reacts with ROS in IBD,[123] convincing evidence that the ability of 5-ASA and SASP to act as scavengers of ROS is the basis of their anti-inflammatory properties is still lacking. Attempts to correlate changes in ROS production at sites of inflammation with antioxidant effects will be difficult for, as for PG and leukotriene production, changes seen may be more a reflection of changes in numbers of leucocytes present.[123] Nevertheless, if SASP and 5-ASA can be shown to act as scavengers of ROS in IBD and RA, then other drugs that are potential antioxidants may also be beneficial in RA and IBD.[124]

### Cellular and Tissue Effects

*Natural killer (NK) cell activity:* A population of the MN cells that is toxic to epithelial cells has been identified with the same phenotype as natural killer (NK) cells,[125] suggesting that cell mediated cytotoxicity by NK cells contributes to the cellular injury in IBD.[126] Thus a possible mechanism of action of SASP and 5-ASA could be through inhibition of NK cell activity.

SASP and SP inhibited *in vitro* cell mediated cytotoxicity by peripheral blood MN cells and SASP by intestinal MN cells,[126] while SASP therapy reduced *ex vivo* NK cell activity in IBD patients.[127] However, 5-ASA in *in vitro* incubations,[125,126,128] or *in vivo* when given to patients,[127] has little effect on NK activity, and thus the effects of SASP may be due to the SP component.[126] Alternatively, they may be due to the diazo bond of SASP since azodisalicylic, that shares with SASP the possession of a diazo bond, also inhibits NK activity,[125] and may thus be a property of the intact SASP molecule. The effects of SASP on NK cell activity may be indirect, through effects on production of cytokines, but are unlikely to involve the cyclooxygenase pathway as indomethacin had no effect on NK activity at concentrations required to inhibit cyclooxygenase.[125,126] SP accounts for the toxic effect of SASP *in vivo* and has toxic effects *in vivo* and *in vitro* on MN cells (as discussed below). It is thus possible that the effects on NK cell activity are a toxic action and the low NK activity reported in IBD a result of drug treatment rather than the disease process.[129]

Whether NK activity is lower[127] or higher[125] in IBD is unclear and in one study neither SASP nor SP inhibited NK activity *in vitro*. The disparity between the therapeutic efficacy of SASP and its metabolites and their effects on NK activity *in vitro* suggests that NK activity is not a major pathogenic mechanism in UC[125] for 5-ASA is the active molecule in IBD and yet has no effect on NK activity. Although the effects of SASP on NK activity are not relevant to IBD, whether they are relevant in RA depends on whether the intact SASP molecule is the active therapeutic moiety, and on the role of NK activity in RA.

*Suppressor cell activity:* Inflammation is a balance between pro-inflammatory and anti-inflammatory aspects of the immune system. The anti-inflammatory properties of SASP and 5-ASA could result from an effect on MN cells, such as inhibition of antibody synthesis or modification of lymphocyte suppressor or helper cell function leading to down-regulation and suppression of the pro-inflammatory aspects of the immune system.[23–25]

This is supported by inhibition of mitogen-stimulated MN cell antibody secretion by SASP and 5-ASA,[129] and proliferation by SASP[22,23,125,129] *in vitro*. 5-ASA also inhibited mitogen-induced expression of cell surface activation markers,[130] but although 5-ASA inhibited mitogen-induced proliferation in some studies[129,131] but in others it was inactive.[23,25,132] SP also had no effect on MN cell antibody production[133] or proliferation,[23,25,129,132] even when added with 5-ASA suggesting that the intact SASP molecule is the active moiety.[25] Conflictingly, in one study SASP, 5-ASA and SP all inhibited mitogen-induced activation of MN cells from healthy subjects and RA patients *in vitro* suggesting that the metabolites of SASP could also be active under some conditions.[24] The effects seen differed with the mitogen used,[24] and it is possible that differences in the studies above are a result of different mitogens being used.

The effects of SASP on induction of a suppressor cell function might be through effects on PGs, which have been implicated in the regulation of suppressor cell activity. Although co-administration of indomethacin with SASP caused a reduction in the effects of SASP on mitogen induced activation in one study,[129] it did not in others.[129,132,133] Thus it is unclear whether PGs are involved. Other cytokines could also be involved.

SASP and 5-ASA inhibited mitogen activation of cells from IBD patients more than those from healthy subjects,[129] suggesting that such raised pro-immunological responses are susceptible to inhibition. In RA, SASP treatment reduced circulating levels of activated lymphocytes and abnormal *ex vivo* lymphocyte responses to mitogens.[24,135] However, in the latter study the change occurred only in those who responded to treatment[135] and thus may have been due to a general reduction in disease activity rather than a direct effect of SASP.[135] Neither SASP, 5-ASA nor SP had any effect on suppressor cell activity when measured directly[136] or on MN cell subpopulations *in vitro* or *in vivo*,[128] and the ability of SASP to inhibit mitogen stimulation of MN cells activation may be due to a toxic effect.[133]

SASP could act therapeutically in IBD and RA by affecting suppressor cell function, with SP being inactive due to its lack of effect. However, 5-ASA is largely inactive in this system and yet is the active component in IBD. Thus if this effect of SASP occurs in patients, then the therapeutic mechanism of action of SASP and 5-ASA may be different. Alternatively, SASP may be toxic to MN cells, as indicated below and the effects seen *in vitro* irrelevant to either IBD or RA.

*Toxic effects:* The few clinical side effects that SASP possesses are associated with the SP component, rather than 5-ASA, although recent studies have suggested that 5-ASA may be nephrotoxic. However, *in vitro* SASP is toxic to MN cells[38,125,133] and other cells,[125] and 5-ASA is toxic to MN cells[24,38] and we have shown the toxicity of SASP and 5-ASA to be dose-dependent.[38] However, other studies have not found SASP[23–25,137] or 5-ASA[125,133,137,138] to be toxic to MN cells, and neither SASP nor 5-ASA to be toxic to PMN,[93] erythrocytes[137] or mouse spleen cells[25] *in vitro.* Additionally 5-ASA was not found to be toxic to other cells in culture.[139]

SASP produces chromosomal damage, namely sister chromatid exchange and micronuclei, to MN cells *in vivo* and *in vitro*, an effect caused by the SP component.[140] However, when the toxicity of SP has been studied neither we[38] nor others have found SP to be toxic in *in vitro* incubations of MN cells,[23,24,133,138] PMN[93] or mouse spleen cells.[25] The toxic side effect of SP *in vivo* may be due to a toxic metabolite rather than SP itself.[137] SASP and 5-ASA may not be toxic *in vivo* due to the rapid acetylation of these compounds once absorbed. Some of the *in vitro* toxic side effects of SASP in MN cells could arise through interference with folate metabolism.[141]

The reason why SASP and 5-ASA are toxic in some studies, but not in others could be due to differences in the incubation conditions, for example the length of time the cells are exposed to the drug. Cell death, leading to lysis and disintegration of cells, is not detected by techniques measuring membrane permeability, such as trypan blue exclusion. The toxic effect at high concentrations of SASP and 5-ASA may indicate more subtle effects on cellular metabolism at lower concentration. If the ability of SASP to inhibit mitogen-stimulated MN cell activation is due to a toxic effect,[129] then some of the other reported effects of SASP and 5-ASA on cells in culture may also be a result of their toxicity.

Thus, SASP and 5-ASA can affect the viability of cells in culture, suggesting that 5-ASA and SASP may possess toxic properties *in vitro*, although there are inconsistencies between these studies. Reliable and sensitive measurement of cell viability may confirm that some of the reported effects of these drugs are due to their toxicity, rather than being physiologically relevant.

### Other Anti-inflammatory Properties

In addition to those discussed above SASP and 5-ASA possess other properties that might explain their anti-inflammatory effects in RA and IBD. These include effects of both 5-ASA and SASP, and not SP, such as the inhibition of synthesis of pro-inflammatory platelet activating factor (PAF),[142] or the inhibition of monocyte-dependent increases in synthesis of $\alpha_1$-acid-glycoprotein,[143] an acute phase protein with anti-inflammatory properties. Others are properties possessed only by 5-ASA, such as inhibition of IL-1$\beta$ production[138] or the reduction of interferon-$\gamma$ induced expression of the HLA-DR major histocompatibility complex on cells.[139] An immunomodulatory function of SASP is also suggested by its beneficial effects in an experimental model of autoimmune disease,[144] its ability to prolong rat cardiac allographs[145] and suppress rejection of intestinal tumours,[146] mediated by suppression of antibody synthesis.[146]

SASP,[114,147,148] and 5-ASA[147,148] and SP[114] all inhibit leucocyte motility, although in some studies 5-ASA[114] and SP[147] had no effect. SASP, but not SP, also inhibits release of leucocyte granules[110,114] that contain various pro-inflammatory agents. Azodisalicylic acid also inhibited granule release,[110] whereas 5-ASA is relatively inactive,[110,112,114] suggesting that this may be a property of the diazo bond possessed by the intact SASP. Thus it is unlikely that this is relevant to clinical effects of both 5-ASA and SASP.

We have shown SASP to inhibit TNF-induced adhesion molecule expression on leucocytes,[149] although this may be due to effects on the receptor for TNF as SASP inhibits the binding of TNF to its receptor.[150] However, neither 5-ASA nor SP affects binding of TNF. Further studies on the effects of 5-ASA and SP on adhesion molecule are required to determine the mechanism of action of SASP on adhesion molecule expression. SASP also inhibits binding of f-mlp to its receptor on leucocytes[151] and thus many of the effects of SASP *in vitro* may be through inhibiting activation of cells by preventing binding of the activating agent. SASP and 5-ASA are highly soluble in ionic and hydrophobic environments and it is possible that they bind to proteins on the surface of cells in a nonspecific fashion. If this is so then it is unlikely to be the basis of their action *in vivo*, given the large number of possible binding sites and proteins with which they will come into contact.

SASP may affect other cells besides the leucocytes. SASP inhibits histamine release from mast cells,[152] and this is suggested to be the basis of its anti-ulcer effects.[153] Although the effects of SASP and 5-ASA on platelet $TXA_2$[42] and PAF[142] production may be due to an effect on platelet function, 5-ASA had no affect on platelet aggregation and fibrinolytic activity either *in vitro* or *in vivo*.[154]

There is as yet insufficient information on the above properties of SASP and 5-ASA to suggest which might be a likely mechanism of action. The effects of the drugs above might be indirect and mediated through effects on production of cytokines such as PGs or leukotrienes, or antioxidant effects increasing the stability of cell membranes.

### Summary

The results of the many *in vitro* and *in vivo* studies performed suggest that there are several possible mechanisms that may explain the anti-inflammatory properties of SASP and 5-ASA. However, it is more probable that 5-ASA and SASP act in IBD by a single mechanism, rather than by several different mechanisms, and that some of the observations are erroneous due to artefactual effects, or misinterpretation of the results. If 5-ASA is the active moiety in IBD and RA then the effects seen with intact SASP molecule are likely to be irrelevant unless they are due to the 5-ASA component within the intact molecule. However, if the intact molecule is the active moiety in RA then SASP and 5-ASA could act by two different anti-inflammatory mechanisms, for although 5-ASA is as good as SASP in the treatment of IBD it has not been shown to be better. If SP acted as an anti-inflammatory agent in RA then it should also do so in the inflamed bowel, where the concentrations are higher. That it does not suggests that the only possible mechanism for SP is as an antibacterial agent in RA. This may suggest that effects seen with SASP or 5-ASA that are also seen with SP may be artefactual. The conflicting findings in some of the studies suggest that the methodology employed in *in vitro* studies can affect the results seen.

The result of studies of the effects of SASP and 5-ASA on the inflammatory changes in patients, either measured *ex vivo* in studies of cells and tissues or *in vivo*, are compounded by the heterogeneous nature of the patient population, such as drug treatments and the site and severity of the inflammation. It is also difficult to determine whether the measured parameter is due to a direct action of the drug or secondary to a change in the activity of the inflammation. As discussed above with respect to PGs the contribution of the leucocyte infiltrate in samples of inflamed tissue used for *ex vivo* and *in vivo* studies is often overlooked, as are the traumatic effects of isolation and preparation procedures on activation of such cells. Animal models are useful in studying the mechanism of action of compounds like 5-ASA, but they do not mimic chronic IBD. A protective effect of SASP and 5-ASA in animal models may be mediated by intervention at one of several points in the inflammatory process, and an effectiveness equal to that of other agents does not necessarily imply a common mechanism. Studies of 5-ASA and SASP in models of RA are still required to dissect out the active component of SASP. If 5-ASA and other compounds act upon mechanisms that are unique to the origin of IBD then the use of such models to determine the mechanism of action of SASP and 5-ASA, and for the development of new anti-inflammatory agents, may be severely limited.

Another problem in studying the mechanism of action of 5-ASA and SASP using *in vitro* models is the relevant concetration of drug that should be used. This is important for, as demonstrated with PG synthesis, opposite results can be obtained using low concentrations compared to those obtained using high concentrations. Furthermore the effective concentrations of 5-ASA and SASP in one anti-inflammatory mechanism are often different to those required for another. For example, it has been suggested that the concentrations achieved of 5-ASA *in vivo* are not high enough to compete with biological material for the ROS, the hydroxyl radical.[106] Which is the relevant concentration is unknown. There is a rapid gradient of drug concentration from the bowel lumen to the perfusing blood, which, due to its poor absorption, will be much steeper for SASP than 5-ASA. Moreover, once absorbed the drugs are rapidly inactivated through acetylation. The high concentrations of 5-ASA and SASP that exist in the bowel lumen are often quoted as justification of the use of similar high concentrations *in vitro* but there is no evidence that 5-ASA and SASP exert their therapeutic effect in the bowel lumen. In IBD although the majority of evidence points to a local action of 5-ASA this is likely to be sited within the colonic mucosa where concentrations will be lower. The effective concentration in RA will be much lower due to rapid metabolism of SASP and 5-ASA, thus implying that high concentrations are not required for the mechanism of action.

The most relevant concentrations will be those at the active site and if this is in the interstitial fluid then it could be relatively high. If it is within a specific cell type, and at a specific organelle then it could be much lower. The ability of SASP to become concentrated in connective tissue where it is then broken down will also produce relatively high, but highly localized concentrations of 5-ASA. The effective concentration will also depend on the activity of the mechanism against which it is acting. Relative concentrations and incubation conditions used in *in vitro* models are designed more with regard to the detection system used to measure the products formed than to reproducing accurately the *in vivo* levels. Thus a high concentration of drug required to have an effect *in vitro* does not necessarily indicate that a similarly high concentration will be required *in vivo* where the activity of the system against which the drug is acting may be much lower. It may thus be more important to consider the relative concentration of the drug compared to the activity of potential target system *in vitro* to that *in vivo*, rather than just drug concentrations.

It is possible that a single property of SASP and 5-ASA explains many of their observed actions. For instance, SASP and 5-ASA can act as antioxidants and prevent lipid peroxidation. The production of PGs also involves ROS and PG synthesis is activated by low levels of peroxides, but is inhibited by high levels. Thus 5-ASA and SASP may affect PG production either by reacting with free radical intermediates in the cyclooxygenase enzyme or by altering levels of lipid peroxides. Similarly, the lipoxygenase pathway involves ROS and the antioxidant glutathione while many of the products resemble those produced by inorganic ROS generated lipid peroxidation. Thus 5-ASA and SASP could also affect this pathway through their scavenging properties. ROS have also been suggested to act as chemical messengers[148] and thus other effects of SASP and 5-ASA could also be due to the effects on ROS. The mechanism by which 5-ASA inhibited the myeloperoxidase activity through scavenging the haemoprotein-associated radical by acting as an alternative substrate[108] is similar to the effects of 5-ASA in PG synthesis and thus may be a general property of these compounds in haem based proteins. This could also explain the effects of SASP and 5-ASA in haemoglobin-induced lipid peroxidation.

Lower levels of von Willebrand factor in patients on SASP[156] suggest that SASP and/or 5-ASA have effects on endothelial cell function in IBD. The early studies by Dr Svartz and her colleagues showed SASP to be able to bind to vascular tissues. The endothelium is also a potent producer of PGs and ROS and has been demonstrated to affect vascular PG production and function. The endothelium is also important in controlling the cellular damage seen in inflammation and ischaemia/reperfusion injury. Thus the protective effects of 5-ASA as an antioxidant might be mediated by the protection of the endothelium and maintenance of production of vascular prostacyclin and other protective compounds. In addition 5-ASA and SASP both have effect on leukotrienes and adhesion molecules which are both important in the regulation of the recruitment of leukocytes through the vascular endothelium and into inflamed sites. Thus whatever the mechanism the vascular endothelium may be the possible site of the anti-inflammatory properties of 5-ASA and SASP.

An understanding of the mechanisms of action of 5-ASA and SASP, when eventually obtained, will be a great asset in the development of new anti-inflammatory agents and may even lead to a deeper understanding of the pathogenesis of RA and IBD. More research is required into the mechanism of action of SASP and 5-ASA in IBD and RA before this can be achieved.

### References

1. Gupta AK, Ellis CN, Siegel MT, *et al.* Sulfasalazine improves psoriasis. *Arch Dermatol* 1990; **126**: 487–493.
2. Svartz N. Sulphasalazine: II. Some notes on the discovery and development of Salazopyrin. *Am J Gastroenterol* 1988; **83**: 497–503.
3. Svartz N. Treatment of ulcerative colitis with salazopyrin. *Int Surg* 1968; **50**: 421–427.
4. Helander S. On the concentration of some sulfanilamide derivatives in different organs and tissue structures: a histo-pharmacological study by means of fluorescence microscopy with some aspects on the possibilities of obtaining high concentrations of chemo-therapeutics in certain tissues. *Acta Physiol Scand* 1945; **Suppl 29**: 1–103.
5. Svartz N. The treatment of rheumatic polyarthritis with acid azo compounds. *Rheumatism* 1948; **4**: 180–185.
6. Svartz N. Salazopyrin, a new sulfanilamide preparation. *Acta Med Scand* 1942; **110**: 577–598.
7. Hanngren A, Hansson E, Svartz N, Ullberg S. Distribution and metabolism of salicyl-azo-sulfapyridine. II. A study with $S^{35}$-salicyl-azo-sulfapyridine and $S^{35}$-sulfapyridine. *Acta Med Scand* 1963; **133**: 391–399.
8. Sinclair RJG, Duthie JJR. Salazopyrin in the treatment of rheumatoid arthritis. *Ann Rheum Dis* 1948; **8**: 226–231.
9. Bargen JA. Symposium on gastro-intestinal conditions; treatment of ulcerative colitis with salicyl azosulfapyridine (Salozopyrin). *Med Clin North Am* 1949; **33**: 935–942.
10. Baron JH, Connel AM, Lennard-Jones JE, Avery Jones F. Sulphasalzine and salicylazosulphadimidine in ulcerative colitis. *Lancet* 1962; **i**: 1094–1096.
11. Misiewicz JJ, Lennard-Jones JE, Connell AM, Baron JH, Avery Jones F. Controlled trial of sulphasalazine in maintenance therapy for ulcerative colitis. *Lancet* 1965: **i**: 185–188.
12. McConkey B, Amos RS, Butler EP, Crockson RA, Crockson AP, Walsh L. Salazopyrin in rheumatoid arthritis. *Agents Actions* 1978; **8**: 438–441.
13. Grindulis KA, McConkey B. Outcome of attempts to treat rheumatoid arthritis with gold, penicillamine, sulphasalazine, or dapsone. *Ann Rheum Dis* 1984; **43**: 398–401.
14. Azad Kahn AK, Piris J, Truelove SC. An experiment to determine the active therapeutic moiety of sulphasalazine. *Lancet* 1977; **ii**: 892–895.
15. Klotz U, Maier K, Fischer C, Heinkel K. Therapeutic efficacy of sulfasalazine and its metabolites in patients with ulcerative colitis and Crohn's disease. *N Engl J Med* 1980; **303**: 1499–1502.
16. Campieri M, Lanfranchi GA, Bazzocchi G, *et al.* Treatment of ulcerative colitis with high-dose 5-aminosalicylic acid enemas. *Lancet* 1981; **ii**: 270–271.
17. Van Hees PAM, Bakker JH, Van Tongeren JHM. Effect of sulphapyridine,

5-aminosalicylic acid and placebo in patients with idiopathic proctitis: a study to determine the active therapeutic moiety of sulphasalazine. *Gut* 1980; **21**: 632–635.
18. Neumann VC, Taggart AJ, LeGallez P, Astbury C, Hill J, Bird HA. A study to determine the active moiety of sulphasalazine in rheumatoid arthritis. *J Rheumatol* 1986; **13**: 285–287.
19. Pullar T, Hunter JA, Capell HA. Which component of sulphasalazine is active in rheumatoid arthritis. *Br Med J* 1985; **290**: 1535–1538.
20. Pullar T, Hunter JA, Capell HA. Sulphasalazine in rheumatoid arthritis: a double blind comparison of sulphasalazine with placebo and sodium aurothiomalate. *Br Med J* 1983; **287**: 1102–1104.
21. Neumann VC, Grindulis KA, Hubball S, McConkey B, Wright V. Comparison between penicillamine and sulphasalazine in rheumatoid arthritis: Leeds—Birmingham trial. *Br Med J* 1983; **287**: 1099–1102.
22. Neumann VC, Grindulis KA. Sulphasalazine in rheumatoid arthritis: an old drug revived (Editorial). *J R Soc Med* 1984; **77**: 169–172.
23. Comer SS, Jasin HE. *In vitro* immunomodulatory effects of sulphasalazine and its metabolites. *J Rheumatol* 1988; **15**: 580–586.
24. Symmons DPM, Salmon M, Farr M, Bacon PA. Sulphasalazine treatment and lymphocyte function in patients with rheumatoid arthritis. *J Rheumatol* 1988; **15**: 575–579.
25. Sheldon P, Webb C, Grindulis KA. Effect of sulphasalazine and its metabolites on mitogen induced transformation of lymphocytes—clues to its clinical action. *Br J Rheumatol* 1988; **27**: 344–349.
26. Gould SP. Prostaglandins, ulcerative colitis and sulphasalazine. *Lancet* 1975; **2**: 988.
27. Smith PR, Dawson DJ, Swan CHJ. Prostaglandin synthetase activity in acute ulcerative colitis: effects of treatment with sulphasalazine, codeine phosphate and prednisolone. *Gut* 1978; **20**: 802–805.
28. Hawkey CJ, Karmeli F, Rachmilewitz D. Imbalance of prostacyclin and thromboxane synthesis in Crohn's disease. *Gut* 1983; **24**: 881–885.
29. Harris DW, Smith PR, Swan CHJ. Determination of prostaglandin synthetase activity in rectal biopsy material and its significance in colonic disease. *Gut* 1978; **19**: 875–877.
30. Ligumsky M, Karmeli F, Sharon P, Zor U, Cohen F, Rachmilewitz D. Enhanced thromboxane $A_2$ and prostacyclin production by cultured rectal mucosa in ulcerative colitis and its inhibition by steroids and sulphasalazine. *Gastroenterology* 1981; **81**: 441–449.
31. Sharon P, Ligumsky M, Rachmilewitz D, Zor U. Role of prostaglandins in ulcerative colitis. Enhanced production during active disease and inhibition by sulphasalazine. *Gastroenterology* 1978; **75**: 638–640.
32. Gould SR. Assay of prostaglandin-like substances in faeces and their measurement in ulcerative colitis. *Prostaglandins* 1976; **11**: 489–497.
33. Gould SR, Brash AR, Conolly HE, Lennard-Jones JE. Studies of prostaglandins and sulphasalazine in ulcerative colitis. *Prostaglandins Leukotrienes Med* 1981; **6**: 165–182.
34. Rampton DS, Sladen GE, Youlten LJF. Rectal mucosal prostaglandin $E_2$ release and its relation to disease activity, electrical potential difference, and treatment in ulcerative colitis. *Gut* 1980; **21**: 591–596.
35. Lauritsen K, Hansen J, Bytzer P, Bukhave K, Rask-Madsen J. Effects of sulphasalazine and disodium azodisalicylate on colonic $PGE_2$ concentrations determined by equilibrium *in-vivo* dialysis of faeces in patients with ulcerative colitis and healthy controls. *Gut* 1984; **25**: 1271–1278.
36. Lauritsen K, Staerk Laursen L, Bukhave K, Rask-Madson J. Use of colonic eicosanoid concentrations as predictors of relapse in ulcerative colitis: double blind placebo controlled study on sulphasalazine maintenance treatment. *Gut* 1988; **29**: 1316–1321.
37. Lauritsen K, Staerk Laursen L, Bukhave K, Rask-Madsen J. Longterm olsalazine treatment: pharmacokinetics, tolerance and effects on local eicosanoid formation in ulcerative colitis and Crohn's colitis. *Gut* 1988; **29**: 974–982.
38. Punchard NA, Boswell DJ, Greenfield SM, Thompson RPH. The effects of sulphasalazine and its metabolites on prostaglandin production by human mononuclear cells. *Biochem Pharmacol* 1992; (in press).
39. Hoult JRS, Page H. 5-aminosalicylic acid: a co-factor for colonic prostacyclin synthesis? *Lancet* 1981; **ii**: 255.
40. Rachmilewitz D, Lugumsky M, Haimovitz A, Treves AJ. Prostanoid synthesis by cultured peripheral blood mononuclear cells in inflammatory diseases of the bowel. *Gastroenterology* 1982; **82**: 673–679.
41. Hawkey CJ, Truelove SC. Inhibition of prostaglandin synthetase in human rectal mucosa. *Gut* 1983; **24**: 213–217.
42. Stenson WF, Lobos E. Inhibition of platelet thromboxane synthetase by sulfasalazine. *Biochem Pharmacol* 1983; **32**: 2205–2209.
43. Hawkey CJ, Boughton-Smith NK, Whittle BJR. Modulation of human colonic arachidonic acid metabolism by sulfasalazine. *Dig Dis Sci* 1985; **30**: 1161–1165.
44. Berry CN, Hoult JRS. Stimulators and inhibitors of prostacyclin formation identified by using intact fragments of rat caecum. *Br J Pharmacol* 1984; **81**: 69P.
45. Kolassa M, Becher R, Wiener H. Influence of sulfasalazine, 5-aminosalicylic acid and sulfapyridine on prostanoid synthesis and metabolism in rabbit colonic mucosa. *Prostaglandins* 1985; **29**: 133–142.
46. Keating JJ, Maxwell WJ, Hogan FP, Keeling PWN. Effects of prednisolone, sulphasalazine, 5-aminosalicylic acid and indomethacin on prostaglandin $E_2$ and leucotriene $B_4$ production. *Gut* 1988; **28**: A1390.
47. Collier HOJ, Francis AA, McDonald-Gibson WJ, Saeed SA. Inhibition of prostaglandin biosynthesis by sulphasalazine and its metabolites. *Prostaglandins* 1976; **11**: 219–225.
48. Hoult JRS, Moore PK. Effects of sulphasalazine and its metabolites on prostaglandin synthesis, inactivation and actions on smooth muscle. *Br J Pharmacol* 1980; **68**: 719–730.
49. Hoult JRS, Moore PK. Sulphasalazine is a potent inhibitor of prostaglandin 15-hydroxydehydrogenase: possible basis for therapeutic action in ulcerative colitis. *Br J Pharmacol* 1978; **64**: 6–8.
50. Hawkey CJ, Boughton Smith NK, Whittle BJR. Comparison of sulphasalazine and benzylimidazole as inhibitors of thromboxane synthesis in human colonic mucosa. *Gastroenterology* 1984; **86**: 1109.
51. Vilaseca J, Salas A, Guarner F, Rodriguez R, Malagelada JR. Participation of thromboxane and other eicosanoid synthesis in the course of experimental inflammatory colitis. *Gastroenterology* 1990; **98**: 269–277.
52. Schlenker T, Peskar BM. Dual effect of sulphasalazine on colonic prostaglandin synthetase. *Lancet* 1981; **ii**: 815.
53. Maxwell WJ, Bloomfield FJ, Hogan FP, Walsh JP, Kelleher D, Keeling PWN. Inhibition of $PGE_2$ secretion by salazopyrin and prednisolone in normal and Crohn's disease monocytes. *Gut* 1985; **26**: A1121.
54. Greenfield SM, Boswell D, Punchard NA and Thompson RPH. Effects of acetyl 5-aminosalicylic acid on lipid peroxidation and prostaglandin production. *Gut* 1990; **31**: A595.
55. Zipser RD, Nast CC, Lee M, Kao HW, Duke R. *In vivo* production of leukotriene $B_4$ and leukotriene $C_4$ in rabbit colitis. Relationship to inflammation. *Gastroenterology* 1987; **92**: 33–39.
56. Kuehl FA, Egan RW. Prostaglandins, arachidonic acid and inflammation. *Science* 1980; **210**: 978–984.
57. Zipser RD, Patterson JB, Kao HW, Hauer CJ, Locke R. Hypersensitive prostaglandin and thromboxane response to hormones in rabbit colitis. *Am J Physiol* 1985; **249**: 457–463.
58. Pacheco S, Hillier K, Smith C. Increased arachidonic acid levels in phospholipids of human colonic mucosa in inflammatory bowel disease. *Clin Sci* 1987; **73**: 361–364.
59. Nishida T, Miwa H, Shigematsu A, Yamamoto M, Iida M, Fujishima M. Increased arachidonic acid composition of phospholipids in colonic mucosa from patients with active ulcerative colitis. *Gut* 1987; **28**: 1002–1007.
60. Rampton DS, Sladen GE. Prostaglandin synthesis inhibitors in ulcerative colitis: flurbiprofen compared with conventional treatment. *Prostaglandins* 1981; **21**: 417–425.
61. Gilat T, Ratan J, Rosen P, Peled Y. Prostaglandins and ulcerative colitis. *Gastroenterology* 1979; **76**: 1083.
62. Rampton DS, McNeil NI, Sarner M. Analgesic ingestion and other factors preceding relapse in ulcerative colitis. *Gut* 1983; **24**: 187–189.
63. Campieri M, Lanfranchi GA, Bazzocchi G, *et al.* Salicylate other than 5-aminosalicylic acid is ineffective in ulcerative colitis. *Lancet* 1978; **ii**: 993.
64. Rampton DS, Sladen GE. Relapse of ulcerative proctocolitis during treatment with non-steroidal anti-inflammatory drugs. *Postgrad Med J* 1981; **57**: 297–299.
65. Allgayer H, Deschryver K, Stenson WF. Treatment with 16,16-dimethyl prostaglandin $E_2$ before and after induction of colitis with trinitrobenzenesulfonic acid in rats decreases inflammation. *Gastroenterology* 1989; **96**: 1290–1300.
66. Robert A, Bundy GL, Field SO, *et al.* Prevention of cecitis in hamsters by certain prostaglandins. *Prostaglandins* 1985; **29**: 961–981.
67. Schumert R, Nast CC, Zipser RD. Effects of prostaglandins on inflammation and leukotriene $B_4$ in experimental colitis: evidence that prostaglandins are anti-inflammatory. *Gastroenterology* 1987; **92**: 1627.
68. Psaila JV, Myers B, Jones IR, Rhodes J. Effect of prostaglandin $PGE_2$ on alcohol-induced ulceration in the rat colon. *Digestion* 1986; **35**: 224–228.
69. Wallace JL, Whittle BJR, Boughton-Smith NK. Prostaglandin protection of rat colonic mucosa from damage induced by ethanol. *Dig Dis Sci* 1985; **30**: 866–876.
70. Moldeus P, Ross D, Larsson R. Co-oxidation of xenobiotics. *Biochem Soc Trans* 1985; **13**: 847–850.
71. Deckmyn H, Zoju C, Arnout J, *et al.* Partial isolation and function of the prostacyclin regulating plasma factors. *Clin Sci* 1985; **69**: 383–393.
72. Blackwell GJ, Flower RJ, Vane JR. Some characteristics of the prostaglandin synthesising system in rabbit kidney microsomes. *Biochim Biophys Acta* 1985; **398**: 178–190.
73. Carpenter MP. Anti-oxidant effects on the prostaglandin endoperoxide synthetase production profile. *Federation Proc* 1981; **40**: 189–194.
74. Panganamala RV, Sharma HM, Heikkila RE, Geer JC, Cornwell DG. Role of hydroxyl radical scavengers dimethyl sulfoxide, alcohols and methional in the inhibition of prostaglandin biosynthesis. *Prostaglandins* 1976; **11**: 599–607.
75. Schiavon R, Freeman GE, Guidi GC, Perona G, Zatti M, Kakkar VV. Selenium enhances prostacyclin production by cultured endothelial cells: possible explanation for increased bleeding times in volunteers taking selenium as a dietary supplement. *Thrombosis Res* 1984; **34**: 389–396.
76. Hurst JS, Paterson CA, Short CS, Hughes MB. Modulation of ocular eicosanoid biosynthesis by oxidants and anti-oxidants. In: Nigram SK, McBrien DCH, Slater TF, eds. *Eicosanoids, Lipid Peroxides and Cancer.* Berlin: Springer-Verlag, 1988; 101–114.
77. Kent RS, Diedrich SL, Whortos AK. Regulation of vascular prostaglandin synthesis by metabolites of arachidonic acid in perfused rabbit aorta. *J Clin Invest* 1987; **72**: 455–462.

78. Hillier K, Mason PJ, Pacheco S, Smith CI. Ulcerative colitis: effects of sulphasalazine, its metabolites and indomethacin on the ability of human colonic mucosa to metabolise prostaglandins *in vitro*. *Br J Pharmacol* 1982; **76**: 157–161.
79. Berry CN, Hoult JRS, Peers SH, Agback H. Inhibition of prostaglandin 15-hydroxydehydrogenase by sulphasalazine and a novel series of potent analogues. *Biochem Pharmacol* 1983; **32**: 2863–2871.
80. Moore PK, Hoult JRS. Selective actions of aspirin and sulphasalazine like drugs against prostaglandin synthesis and breakdown. *Biochem Pharmacol* 1982; **31**: 969–971.
81. Jarabak J, Brathwaite SS. Kinetic studies on a 15-hydroxy prostaglandin dehydrogenase from human placenta. *Arch Biochem Biophys* 1976; **177**: 245–254.
82. Lin YM, Jarabak J. Isolation of two proteins with 9-ketoprostaglandin reductase and NADP linked 15-hydroxyprostaglandin dehydrogenase activities and studies on their inhibition. *Biochem Biophys Res Comm* 1978; **81**: 1227–1234.
83. Korff J, Jarabak J. Partial isolation and characterization of the 15-hydroxyprostaglandin dehydrogenases and 9-ketoprostaglandin reductases in rabbit kidney. *Prostaglandins* 1980; **20**: 111–125.
84. Jarabak, J, Luncsford A, Berkowitz D. Substrate specificities of three prostaglandin dehydrogenases. *Prostaglandins* 1983; **26**: 849–866.
85. Bakhle YS. Effects of sulphasalazine on prostaglandin inactivation and synthesis in isolated lungs of guinea pig, rat and man. *Eur J Pharmacol* 1980; **68**: 493–496.
86. Hellewell PG, Pearson JD. Effect of sulphasalazine on pulmonary inactivation of prostaglandin $F_{2\alpha}$ in the pig. *Br J Pharmacol* 1982; **76**: 319–326.
87. Allgayer H, Eisenburg J, Paumgartner G. Soybean lipoxygenase inhibition: studies with the sulphasalazine metabolites *N*-acetylaminosalicylic acid, 5-aminosalicylic acid and sulphapyridine. *Eur J Clin Pharmacol* 1984; **26**: 449–451.
88. Sircar JC, Schwender CF, Carethers ME. Inhibition of soybean lipoxygenase by sulphasalazine and 5-aminosalicylic acid: a possible mode of action in ulcerative colitis. *Biochem Pharmacol* 1983; **32**: 170–172.
89. Marcinkiewicz E, Duniec Z, Robak J. Salazosulfapyridine and non-steroidal anti-inflammatory drugs do not inhibit soybean lipoxygenase. *Biochem Pharm* 1985; **34**: 148–149.
90. Nielson OH, Ahnfelt-Ronne I. 4-aminosalicylic acid, in contrast to 5-aminosalicylic acid, has no effect on arachidonic acid metabolism in human neutrophils or on the free radical 1,1-Diphenyl-2-picrylhydrazyl. *Pharmacol Toxicol* 1988; **62**: 223–226.
91. Nielson OH, Bukhave K, Elmgreen J, Ahnfelt-Ronne I. Inhibition of 5-lipoxygenase pathway of arachidonic acid metabolism in human neutrophils by sulfasalazine and 5-aminosalicylic acid. *Dig Dis Sci* 1987; **32**: 577–582.
92. Ahnfelt-Ronne I, Nielson OH, Bukhave K, Elmgreen J. Sulfasalazine and its anti-inflammatory metabolite 5-aminosalicylic acid: effect on arachidonic acid metabolism in human neutrophils, and free radical scavenging. *Adv Prostaglandin Thromboxane Leukotriene Res* 1987; **17B**: 918–922.
93. Stenson WF, Lobos E. Sulfasalazine inhibits the synthesis of chemotactic lipids by neutrophils. *J Clin Invest* 1982; **69**: 494–497.
94. Nielson ST, Beninati L, Buonato CB. Sulfasalasine and 5-aminosalicylic acid inhibit contractile leukotriene formation. *Scand J Gastroenterol* 1988; **23**: 272–276.
95. Sharon P, Stenson WF. Enhanced synthesis of leukotriene $B_4$ by colonic mucosa in inflammatory bowel disease. *Gastroenterol* 1984; **86**: 454–460.
96. Dreyling KW, Hoppe U, Peskar BA, Schaarschmidt K, Peskar BM. Leukotrienes in Crohn's disease: effect of sulfasalazine and 5-aminosalicylic acid. *Adv Prostaglandin Thromboxane Leukotriene Res* 1987; **17**: 339–343.
97. Peskar BM, Dreyling KW, May B, Schaarschmidt K, Goebell H. Enhanced formation of sulfidopeptide leukotrienes in ulcerative colitis and Crohn's disease: inhibition by sulphasalazine and 5-aminosalicylic acid. *Agents Actions* 1986; **18**: 381–383.
98. Peskar BM, Dreyling KW, May B, Schaarschmidt K, Goebell H. Possible mode of action of 5-aminosalicylic acid. *Dig Dis Sci* 1987; **32** (12 suppl): 51S–56S.
99. Bach MK, Brashler JR, Johnson MA. Inhibition by sulfasalazine of LTC synthetase and of rat liver glutathione S-transferases. *Biochem Pharmacol* 1985; **34**: 2695–2704.
100. Wallace JL, MacNaughton WK, Morris GP, Beck PL. Inhibition of leukotriene synthesis markedly accelerates healing in a rat model of inflammatory bowel disease. *Gastroenterology* 1989; **96**: 29–36.
101. Hawkey CJ, Rampton DS. Benoxoprofen in the treatment of active ulcerative colitis. *Prostaglandins Leukotrienes Med* 1983; **10**: 405–409.
102. Staerk Laursen L, Naesdal J, Bukhave K, Lauritsen K, Rask-Madsen J. Selective 5-lipoxygenase inhibition in ulcerative colitis. *Lancet* 1990; **335**: 683–685.
103. Aruoma OI, Wasil M, Halliwell B, Hoey BM, Butler J. The scavenging of oxidants by sulphasalazine and its metabolites. A possible contribution to their anti-inflammatory effects. *Biochem Pharmacol* 1987; **36**: 3739–3742.
104. Dull BJ, Salata K, Langenhove AV, Goldman P. 5-aminosalicylate: oxidation by activated leukocytes and protection of cultured cells from oxidative damage. *Biochem Pharmacol* 1987; **36**: 2467–2472.
105. Miyachi Y, Yoshioka A, Imamura S, Niwa Y. Effect of sulphasalazine and its metabolites on the generation of reactive oxygen species. *Gut* 1987; **28**: 190–195.
106. Hallwell B. Free radical scavengers and inflammatory bowel disease. *Gastroenterology* 1991; **101**: 872.
107. Kvietys PR, Morgan-Smith S, Grisham MB, Manci EA. 5-Aminosalicylic acid protects against ischemia/reperfusion-induced gastric bleeding in the rat. *Gastroenterology* 1988; **94**: 733–738.
108. Halliwell B. Sulphasalazine and oxidant scavenging in ulcerative colitis. *Lancet* 1987; 635.
109. Von Ritter C, Grisham MB, Granger DN. Sulfasalazine metabolites and dapsone attenuate formyl-methionyl-leucyl-phenylalanine-induced mucosal injury in rat ileum. *Gastroenterology* 1989; **96**: 811–816.
110. Neal TM, Winterbourn CC, Vissers MCM. Inhibition of neutrophil degranulation and superoxide production by sulfasalazine: comparison with 5-aminosalicylic acid, sulfapyridine and olsalazine. *Biochem Pharmacol* 1987; **36**: 2765–2768.
111. Williams JG, Hallett MB. Effect of sulphasalazine and its active metabolite, 5-aminosalicylic acid on toxic oxygen metabolite production by neutrophils. *Gut* 1989; **30**: 1581–1587.
112. Dallegri F, Ottonello L, Ballestrero A, Bogliolo F, Ferrando F, Patrone F. Cytoprotection against neutrophil derived hypochlorous acid: a potential mechanism for the therapeutic action of 5-aminosalicylic acid in ulcerative colitis. *Gut* 1990; **31**: 184–186.
113. Kanerud L, Hafstrom I, Ringertz B. Effect of sulphasalazine and sulphapyridine on neutrophil superoxide production: role of cytosolic free calcium. *Ann Rheum Dis* 1990; **49**: 296–300.
114. Molin L, Stendahl O. The effect of sulfasalazine and its active components on human polymorphonuclear leukocyte function in relation to ulcerative colitis. *Acta Med Scand* 1979; **206**: 451–457.
115. Craven PA, Pfansteil J, Saito R, DeRubertis FR. Actions of sulfasalazine and 5-aminosalicylic acid as reactive oxygen scavengers in the suppression of bile acid-induced increases in colonic epithelial cell loss and proliferative activity. *Gastroenterology* 1987; **92**: 1998–2008.
116. Greenfield SM, Punchard NA, Thompson RPH. Inhibition of red cell membrane lipid peroxidation by sulphasalazine and 5-aminosalicylic acid. *Gut* 1991; **32**: 1156–1159.
117. Yamada T, Volkmer C, Grisham MB. The effects of sulfasalazine metabolites on hemoglobin-catalysed liquid peroxidation. *Free Radic Biol Med* 1991; **10**: 41–49.
118. Ahnfelt-Ronne I, Nielson OH. The anti-inflammatory moiety of sulfasalasine, 5-aminosalacilic acid, is a radical scavenger. *Agents Actions* 1987; **21**: 191–194.
119. Keshavazian A, Morgan G, Sedghi S, Gordon JH, Doria M. Role of reactive oxygen metabolites in experimental colitis. *Gut* 1990; **31**: 786–790.
120. Del Soldato P, Campieri M, Brignola G, *et al.* A possible mechanism of action of sulfasalazine and 5-aminosalicylic acid in inflammatory bowel disease. Interactions with oxygen free radicals. *Gastroenterology* 1985; **89**: 1215–1216.
121. Pekoe G, Van Dyke K, Mengoli H, Peden D, English D. Comparison of the effects of antioxidant non-steroidal anti-inflammatory drugs against myeloperoxidase and hypochlorous acid luminol-enhanced chemiluminescence. *Agents and Actions* 1982; **12**: 232–238.
122. Aruoma OI, Halliwell B. The iron-binding and hydroxyl radical scavenging action of anti-inflammatory drugs. *Xenobiotica* 1988; **18**: 459–470.
123. Ahnfelt-Ronne I, Nielson OH, Christensen A, Langholz E, Binder V, Riis P. Clinical evidence supporting the radical scavenger mechanism of 5-aminosalicylic acid. *Gastroenterology* 1990; **98**: 1162–1169.
124. Miyachi Y. Anti-oxidant effects of sulfones and sulfonamides on inflammatory bowel diseases. In: Tsuchiya M, *et al.*, eds. *Free Radicals in Digestive Diseases*. Amsterdam: Elsevier Science Publishers, 1988; 53–55.
125. Gibson PR, Jewell DP. Sulphasalazine and derivatives, natural killer activity and ulcerative colitis. *Clin Sci* 1985; **69**: 177–184.
126. MacDermott RP, Kane MG, Steele LL, Stenson WF. Inhibition of cytotoxicity by sulfasalazine. I. Sulphasalazine inhibits spontaneous cell-mediated cytotoxicity by peripheral blood and intestinal mononuclear cells from control and inflammatory bowel disease patients. *Immunopharmacology* 1986; **11**: 101–109.
127. Aparicio-Pages MN, Verspaget HW, Hafkensheild JCM, *et al.* Inhibition of cell mediated cytotoxicity by sulphasalazine: effect of *in vivo* treatment with 5-aminosalicylic acid and sulphasalazine on *in vitro* natural killer cell activity. *Gut* 1990; **31**: 1030–1032.
128. Thayer WR, Charloid C, Field CE. Effects of sulphasalazine on selected lymphocyte subpopulation *in vivo* and *in vitro*. *Dig Dis Sci* 1979; **24**: 672–679.
129. Holdstock G, Chastney BF, Krawitt EL. Increased suppressor cell activity in inflammatory bowel disease. *Gut* 1981; **22**: 1025–1030.
130. Schrieber S, MacDermot RP, Raedler A, Pinnau R, Bertovich MJ, Nash GS. Increased activation of isolated intestinal lamina propria mononuclear cells in inflammatory bowel disease. *Gastroenterology* 1991; **101**: 1020–1030.
131. Elitsur Y, Freedland C, Luk GD. Inhibition of DNA synthesis in human peripheral and lamina propria lymphocytes by 5-aminosalicylic and hydrocortisone. *Regional Immunol* 1990; **3**: 56–61.
132. Ali ATMM, Basran GS, Morley J. Mode of action of sulphasalazine: an alternative view. *Lancet* 1982; **i**: 506–507.
133. MacDermot RP, Schloemann SR, Bertovich MJ, Nash GS, Peters M and Stenson WF. Inhibition of antibody secretion by 5-aminosalicylic acid. *Gastroenterology* 1989; **96**: 442–448.
134. Hillier K, Jones DB, Pacheco S. The action of sulphasalazine, indomethacin and BW-775C on human peripheral blood mononuclear cell activation by PHA. *Br J Pharmacol* 1984; **81**: 166P.

135. Samanta A, Webb C, Grindulis KA, Sheldon P. Sulphasalazine and lymphoproliferative responses in rheumatoid arthritis. *Clin Sci* 1991; **80** (suppl. 24): 18P–19P.
136. Holdstock G, Chastenay BF, Krawitt EL. Functional suppressor T cell activity in Crohn's disease and the effects of sulphasalazine. *Clin Exp Immunol* 1982; **48**: 619–624.
137. Piromohamed M, Coleman MD, Hussain F, Breckinridge SM, Park BK. Direct and metabolism dependent toxicity of sulphasalazine and its principal metabolites towards human erythrocytes and leucocytes. *Br J Clin Pharmacol* 1991; **32**: 303–310.
138. Mahida YR, Lamming CED, Gallagher A, Hawthorne AB, Hawkey CJ. 5-aminosalicylic acid is a potent inhibitor of interleukin 1$\beta$ production in organ culture of colonic biopsy specimens from patients with inflammatory bowel disease. *Gut* 1991; **32**: 50–54.
139. Crotty B, Hoany P, Dalton HR, Jewel DP. Salicylates used in inflammatory bowel disease and colchicine impair interferon-$\gamma$ induced HLA-DR expression. *Gut* 1992; **33**: 59–64.
140. Mackay JM, Fox DP, Brunt PW, Hawkworth GM, Brown JE. Chromosome damage in human lymphocytes *in vitro*: the effects of sulphasalazine and its sulphapyridine metabolites. *Gut* 1988; **29**: A708.
141. Baum CL, Selhub J, Rosenberg IH. Antifolate actions of sulfasalazine on intact lymphocytes. *J Lab Clin Med* 1981; **97**: 779–784.
142. Eliakim R, Karmeli F, Razin E, Rachmilevitz D. Role of platelet-activating factor in ulcerative colitis: enhanced production during active disease and inhibition by sulfasalazine and prednisolone. *Gastroenterology* 1988; **95**: 1167–1172.
143. Mazlam MZ, Cunningham J, Hodgson HJF. Effect of sulphasalazine and 5-aminosalicylic acid on $\alpha_1$-acid glycoprotein synthesis *in vitro*. *Gut* 1991; **32**: A589.
144. Holmdahl R, Klarenkoy L, Rubin K, *et al.* Role of T lymphocytes in murine collagen induced arthritis. *Agents and Actions* 1987; **19**: 295–305.
145. Wanders A, Tufreson G, Gerdin B. The enhancing effect of cyclosporin A and sulfasalazine on the prevention of rejection in rat cardiac cellographs. *Transplant Int* 1988; **1**: 113–115.
146. Laursen ML. The influence of salicyl-azo-sulfapyridine on the immune response to antigen tumour cells inoculated into the caecal lumen of C3H mice. *Scand J Gastroenterol* 1978; **13**: 991–997.
147. Rhodes JM, Bartholomew TC, Jewell DP. Inhibition of leucocyte motility by drugs used in ulcerative colitis. *Gut* 1981; **22**: 642–647.
148. Nielson OH, Verspaget HW, Elmgreen J. Inhibition of intestinal macrophage chemotaxis to leukotriene $B_4$ by sulphasalazine, olsalazine and 5-aminosalicylic acid. *Aliment Pharmacol Therap* 1988; **2**: 203–211.
149. Greenfield SM, Hamblin AS, Punchard NA, Thompson RPH. Inhibition of leukocyte adhesion molecule expression: a novel mechanism of action of sulphasalazine. *Gut* 1991; **32**: A1228.
150. Shanahan F, Miederlehner A, Carramanzana N, Anton P. Sulfasalazine inhibits the binding of TNF$\alpha$ to its receptor. *Immunopharmacol* 1990; **20**: 217–224.
151. Stenson WF, Mehta J, Spilberg I. Sulphasalazine inhibition of binding of *N*-formyl-methionyl-leucyl-phenylalanine (FMLP) to its receptor on human neutrophils. *Biochem Pharmacol* 1984; **33**: 407–412.
152. Barrett KE, Tashof TL, Metcalfe DD. Inhibition of IgE-mediated mast cell degranulation by sulphasalazine. *Eur J Pharmacol* 1985; **107**: 279–281.
153. Ogle CW, Cho CH. Effects of sulphasalazine on stress ulceration and mast cell degranulation in rat stomach. *Eur J Pharmacol* 1985; **112**: 285–286.
154. Winther K, Bondesen S, Honore-Hansen S, Hvidberg EF. Lack of effect of 5-aminosalicylic acid on platelet aggregation and fibrinolytic activity *in vivo* and *in vitro*. *Eur J Clin Pharmacol* 1987; **33**: 419–422.
155. Saran M, Bors W. Oxygen radicals acting as chemical messengers: a hypothesis. *Free Rad Res Comm* 1987; **7**: 213–220.
156. Nilsson TK, Rutegard JN, Ashgren LR, Stenling RB. Effects of sulfasalazine on plasma levels of the endothelial cell products, von Willebrand factor and tissue plasminogen activator in patients with ulcerative colitis. *Surg Res Comm* 1989; **4**: 285–288.

ACKNOWLEDGEMENTS. We are grateful to the Special Trustees of St Thomas' Hospital and the South East Thames Regional Health Authority for their support. We would like to thank Miss Diane Boswell and Mr Duncan Watson for their help in the preparation of this manuscript.

**Received 14 April 1992; accepted 15 April 1992**
